# Synergistic Enhancement Properties of a Flexible Integrated PAN/PVDF Piezoelectric Sensor for Human Posture Recognition

**DOI:** 10.3390/nano12071155

**Published:** 2022-03-31

**Authors:** Jiliang Mu, Shuai Xian, Junbin Yu, Juanhong Zhao, Jinsha Song, Zhengyang Li, Xiaojuan Hou, Xiujian Chou, Jian He

**Affiliations:** Science and Technology on Electronic Test and Measurement Laboratory, North University of China, Taiyuan 030051, China; xianyu0279@163.com (S.X.); yujunbin@nuc.edu.cn (J.Y.); zhaojuanhong2022@163.com (J.Z.); jinsha2006daxue@163.com (J.S.); 18222586812@163.com (Z.L.); houxiaojuan@nuc.edu.cn (X.H.); chouxiujian@nuc.edu.cn (X.C.)

**Keywords:** PAN/PVDF, integrated structure, synergistic piezoelectricity, flexible pressure sensor, human posture recognition

## Abstract

The flexible pressure sensor has attracted much attention due to its wearable and conformal advantage. All the same, enhancing its electrical and structural properties is still a huge challenge. Herein, a flexible integrated pressure sensor (FIPS) composed of a solid silicone rubber matrix, composited with piezoelectric powers of polyacrylonitrile/Polyvinylidene fluoride (PAN/PVDF) and conductive silver-coated glass microspheres is first proposed. Specifically, the mass ratio of the PAN/PVDF and the rubber is up to 4:5 after mechanical mixing. The output voltage of the sensor with composite PAN/PVDF reaches 49 V, which is 2.57 and 3.06 times that with the single components, PAN and PVDF, respectively. In the range from 0 to 800 kPa, its linearity of voltage and current are all close to 0.986. Meanwhile, the sensor retains high voltage and current sensitivities of 42 mV/kPa and 0.174 nA/kPa, respectively. Furthermore, the minimum response time is 43 ms at a frequency range of 1–2.5 Hz in different postures, and the stability is verified over 10,000 cycles. In practical measurements, the designed FIPS showed excellent recognition abilities for various gaits and different bending degrees of fingers. This work provides a novel strategy to improve the flexible pressure sensor, and demonstrates an attractive potential in terms of human health and motion monitoring.

## 1. Introduction

Wearable electronics, especially the flexible pressure sensor, have great potential applications in the field of internet of things for personalized identification, medical research and human-computer interaction [1,2,3,4,5]. For the flexible wearable pressure sensor, its stability and reliability largely depend on the capability of the external power supply. A traditional power supply using a battery is limited by the compatibility of the rigid materials with skin, the charge decay over a long duty time, and it cannot be widely used in wearable electronics [6]. To overcome these limitations, a self-powered piezoelectric nanogenerator (PENG) [7,8] (all the abbreviations in this article are listed in Table S1), has been rising and becoming the main equipment for sustainable, wearable energy collection, and can effectively convert mechanical energy into electrical energy [9,10] and realize its self-powered work [11]. Based on this, it has been used for monitoring and identifying various posture movements of the human body [12].

There remains a challenge for piezoelectric devices to balance flexibility and electromechanical conversion capability [13,14]. In order to monitor human posture, the electrical output of wearable electronic devices should be easily obtained and observed [15]. Hence, to achieve both high output performance and good flexibility is a huge challenge [16,17]. As an essential part of the PENGs, piezoelectric materials play an important role in improving electric output performance [18]. Currently, a variety of piezoelectric materials have been widely investigated, including lead zirconate titanate [19], BaTiO_3_ [20], ZnO [21], composites [22], polymers [23] and so on. Among them, polymer composites are very popular in wearable electronics because of their high piezoelectric coefficient and good flexibility [24]. Polyvinylidene fluoride (PVDF) as a typical semi-crystalline piezoelectric polymer has been widely used [21], and polyacrylonitrile (PAN) as a new piezoelectric polymer is drawing much attention. However, there are few reports on the piezoelectric properties of PAN and PVDF composites, which is worth exploring for the study of flexible piezoelectric sensors with high performance. Currently, there are still some problems to be solved. First, PAN has higher piezoelectric output and lower dielectric loss compared with PVDF, but its output performance still needs to be enhanced [25]. For this, some researchers have suggested that different piezoelectric materials may produce synergistic piezoelectric effects to improve the output performance of piezoelectric devices [24,26]. Second, it is difficult for the filling ratio of piezoelectric materials to polymer matrix to be improved effectively. In earlier research reports, Sun et al. [24] reported a flexible PENG based on a nanofabric with a filling ratio of 18%. Jiang et al. [27] design a stretchable, breathable and stable nanofiber composite with a filling rate of 12%. The filling ratio of nanofibers prepared by Liu et al. [13] and Cherumannil Karumuthil et al. [28] by electrospinning was 13% and 10%, respectively. Similarly, Yang et al. [29] prepared a flexible piezoelectric pressure sensor with a maximum filling ratio of 21% by magnetic stirring. This can result in poor output performance for devices because of the low fill ratio. Finally, it has been reported that the interface between the electrode layer and piezoelectric layer of a piezoelectric device produces interfacial debonding extremely easily [30,31,32]. This results in a failed whole device and greatly affects the charge transport capability and reliability of the device under the multiple motions of stretching and releasing.

Herein, we developed a new method of mixing PAN and PVDF powder into solid silicone rubber to prepare a flexible integrated pressure sensor (FIPS) with high performance. The mechanical mixing process can significantly improve the filling rate of fillers (PVDF and PAN) to 44% and increase the output performance of the device. In the process of vulcanization, the matrix for the piezoelectric layer and the electrode layer uses the same material, so the two layers can be seamlessly connected and completely integrated as a whole structure, which leads to high flexibility and good adaptability of the device and it will not be damaged during multiple stretching and releasing motions. At the same time, due to the piezoelectric synergistic effect of PAN and PVDF, the piezoelectric properties of the composite films are greatly improved, and the output voltage can reach 49 V. On account of the superiority of the preparation process and materials, the FIPS has good sensitivity, a high linearity and a rapid response time. In addition, the FIPS can be carried and used at any time as a wearable electronic due to its self-powered advantage. As a novel wearable electronic device, it is easily installed on each joint or foot to identify different bending angles of joints and different movements of the human body, so as to realize human posture detection and gait recognition, which is significant for the development of wearable electronics, human–machine interaction, medical testing and personalized identification.

## 2. Experimental Section

### 2.1. Materials

The PAN powder (Mw, 1,500,000) and PVDF powder (Mw, 534,000) were purchased from Hubei Dechao Chemical Co., Ltd. and Sigma–Aldrich, respectively. Silver-coated glass microspheres (with an average diameter of 15~50 μm) were obtained from Shenzhen Changxinda Shielding Material Co. Ltd., Shenzhen, China, and silicone rubber was purchased from Guangzhou Tianan Silicone Rubber Technology Co., Ltd., Guangzhou, China.

### 2.2. Fabrication of the Flexible Integrated Pressure Sensors (FIPSs)

First, the PAN (6.4 g) powder and the PVDF (1.6 g) powder were mixed, then the mixture of the PAN/PVDF powder was added to 10 g silicone rubber (the roller rotational speed and roller spacing were finally set at 140 rad/s and 400 μm, respectively) and stirred for 1.5 h to make the mixture uniformly distributed, thus forming a piezoelectric layer. Similarly, PAN powder or PVDF powder and silicone rubber were mixed according to the 4:5 mass ratio in the same way, respectively. Next, according to the above method, the silver-coated glass microspheres and silicone rubber were mixed according to the mass ratio of 3:1 to make a stretchable electrode. The electrode was then cut into two pieces of the same size and placed on the upper and lower sides of the piezoelectric layer. After the mixing process, the sandwich structure was put into a steel mold (hollow structure, thickness 2 mm) and cured by a high temperature plate vulcanizing machine at 175 °C and 20 MPa pressure for 17 min. Finally, the prepared piezoelectric film was cut into a 3 cm × 3 cm square sample. Iit was poled at 120 °C by applying an electric field of 1.6 kV/mm for 60 min. At this time, the stretchable one-piece piezoelectric self-powered flexible pressure sensor was obtained.

### 2.3. Characterization and Measurement

Fourier-transform infrared (FTIR) of the samples were measured by BRUKER ALPHA II Fourier infrared spectrometer (BRUKER OPTICS, Ettlingen, Germany), and X-ray diffraction (XRD) analysis was carried out using the Rigaku Ultima IV X-ray diffractometer (Rigaku Corporation, Tokyo, Japan). The surface morphologies of the PAN/PVDF composite films were characterized by field emission scanning electron microscopy (SEM, HITACHI SU8020, Tokyo, Japan) and the element distribution was qualitatively tested by using an energy disperse spectrometer (EDS, HORIBA EMAX evolution X-Max 80/EX-270). The composite films were fastened onto the fixed end and 1 × 1 cm^2^ acrylic was fastened onto the active end of the linear motor, in order to realize quantitative measurements. The output performances of voltage and current were measured by a Keithley 2611B system electrometer. At the same time, all data collection and analysis were carried out using the LabVIEW programming interface, which realizes real-time data collection and analysis (Figure S1).

## 3. Results and Discussion

**Figure 1:**
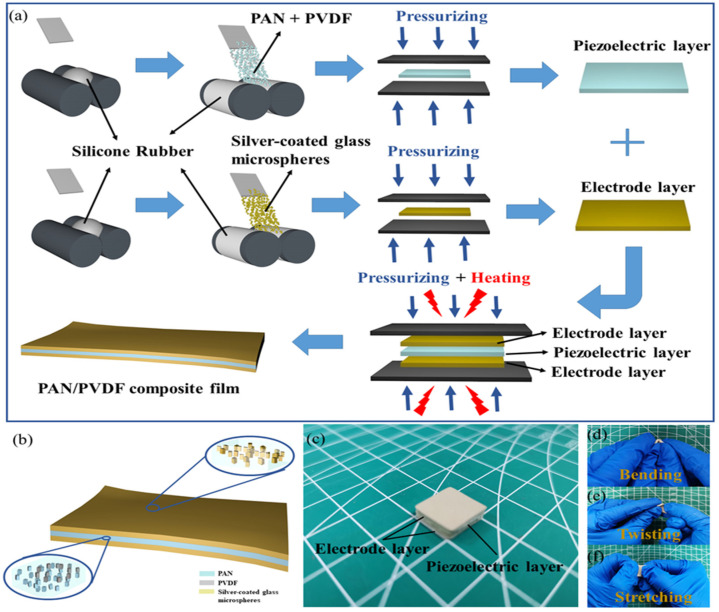
(**a**) Fabrication of the PAN/PVDF composite film. (**b**) Schematic diagram of each functional layer. (**c**) Digital photograph of the FIPS. (**d**–**f**) Photographs of the FIPS under different deformations.

Piezoelectric PAN/PVDF powders and conductive silver-coated glass microspheres were added into the silicon rubber matrix and mixed evenly to obtain the PAN/PVDF composite films and stretchable electric electrodes, respectively. The prepared piezoelectric layer and electrode layer are designed as the sandwich structures and are fabricated as FIPS. The schematic fabrication process is illustrated in Figure 1a. In detail, the structural diagram and digital photograph of the FIPS are shown in Figure 1b,c, respectively. It can be seen that the thickness of each layer is uniform, and the two interfaces between the electrode layer and the piezoelectric layer all show close connection and maintain good adhesion. Regarding its whole flexibility, the FIPS can withstand different deformations. As shown in Figure 1d–f, its piezoelectric layer and electrode layer are still connected perfectly and have good integrity when the FIPS is bent, twisted, and stretched in half, under the action of various external forces.

### 3.1. Piezoelectric Enhanced Mechanism Analysis for PAN/PVDF Composite Films

**Figure 2:**
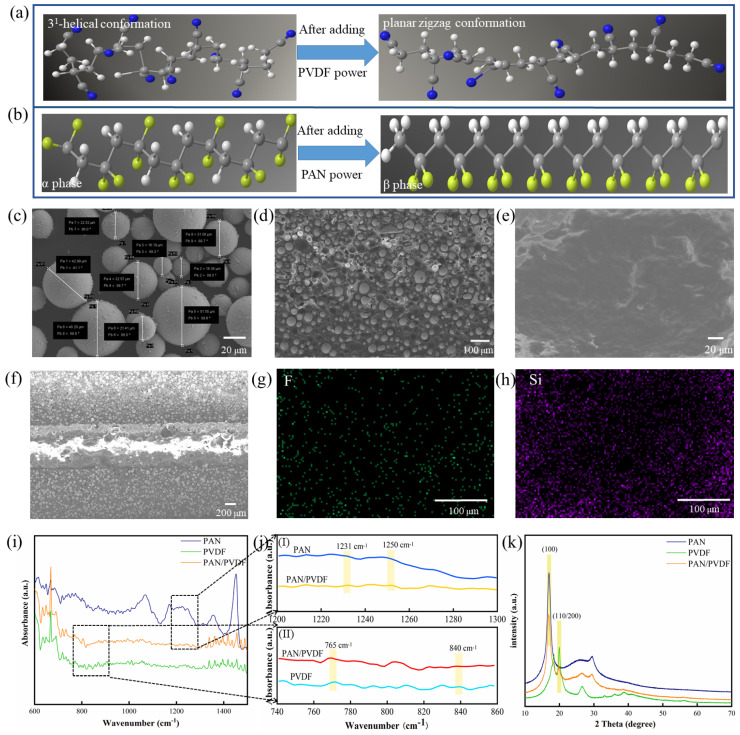
The conformational transition model of (**a**) PAN and (**b**) PVDF. (**c**) The SEM diagram of silver-coated glass microspheres. (**d**) The SEM cross-section image of the electrode layer of the composite film. (**e**) SEM diagram and (**f**) SEM cross-section of the PAN/PVDF composite membrane. (**g**,**h**) EDS spectrum of the PAN/PVDF composite film. (**i**,**j**) FTIR spectra of the PAN, PVDF and PAN/PVDF. (**k**) XRD patterns of PAN, PVDF and PAN/PVDF.

The PVDF unit is CF_2_-CH_2_, and its two fluorine atoms are both on one side and combined with a carbon atom. Since fluorine is more electronegative than hydrogen and carbon atoms, a dipole moment is formed in the unit, so that each main chain has a dipole moment perpendicular to the polymer chain. As a new type of piezoelectric polymer, PAN is an amorphous vinyl-type polymer, which contains cyano(–CN) in each repeating unit, the cyano produces a large dipole moment [33], and PAN has a larger dipole moment than PVDF [34]. In addition, the larger the dipole moment, the better the piezoelectricity of the polymer material. Therefore, PAN has a greater potential for piezoelectric materials. Different molecular conformations of polymer materials correspond to different dipole moments. Among the α, β, γ, δ and ɛ phase of PVDF, β phase (planar zigzag conformation) has the largest dipole moment (2.1 D) and exhibits better piezoelectric properties compared with other conformations [29]. Similarly, PAN mainly has planar zigzag and 3^1^-helical conformations [35], and planar zigzag conformation has better piezoelectric properties [24]. Hence, increasing the conformation content of the planar zigzag conformation is an important way to improve the piezoelectric properties of PAN. The 3^1^-helical conformations of PAN can be transformed into planar zigzag conformation through certain preparation methods or material doping (the conformational transition model of PAN is shown in the Figure 2a), which is beneficial for improving the piezoelectric properties of PAN. At the same time, the piezoelectric properties of PVDF can also be improved by material doping to change the α phase into β phase (Figure 2b). Therefore, the piezoelectric properties of PVDF and PAN can be improved by doping them.

### 3.2. Characterization of the PAN/PVDF Film

Figure 2c,d shows the SEM diagram of the silver-coated glass microspheres and the SEM profile of the electrode layer of the composite film, respectively. It can be clearly observed that the silver-coated glass microspheres (20~50 μm in diameter) are uniformly dispersed in the silicone rubber matrix. The SEM diagram of the PAN/PVDF composite film shows that PAN powder and PVDF powder are uniformly mixed in the matrix (Figure 2e). Figure 2f shows SEM section images of the PAN/PVDF composite film. The cross-section imaging of the interface between the electrode layer and piezoelectric layer shows that the three layers are crosslinked to form a single structure. Thanks to the uniform integration process, and because the electrode layers and piezoelectric layer use the same silicone rubber matrix, they can be seamlessly combined with each other. Furthermore, it can be observed that the piezoelectric phase and the conductive phase are closely and uniformly distributed in the silicone rubber matrix. The energy dispersive spectroscopy (EDS) spectrum of the PAN/PVDF composite film is shown in Figure 2g,h. The results clearly show the uniform distribution of F and Si elements in the sample, which proves that PVDF particles and silicone rubber can be uniformly mixed using the superior mixing process. (Detailed EDS diagrams are shown in Figure S2). Figure 2i shows the FTIR spectra of PAN, PVDF and PAN/PVDF powders. In general, the wavenumbers of 1231 cm^−1^ and 1250 cm^−1^ in pure PAN powder represent the 3^1^-helix structure caused by the distorted vibration of the CH_2_ group, and the planar zigzag conformation caused by the plane rocking vibration of the CH group and the non-planar rocking vibration of the CH_2_ group, respectively [35]. For PVDF, the characteristic α phase absorption peaks were 765 cm^−1^ for CF_2_ bending and 975 cm^−1^ for CH_2_ rocking, while the typical β phase absorption peaks were 840 cm^−1^ (CF_2_ stretching, CH_2_ rocking and skeletal C–C stretching) and 1276 cm^−1^ [36]. Due to the piezoelectric properties, PAN and PVDF are related to the proportion of planar zigzag conformation and β phase respectively. The zigzag conformation content C of PAN and the β phase content F (β) of PVDF can be estimated by equations according to the FTIR spectrum [25,37]:

(1) $$C=\frac{I_{1250}}{I_{1231}},$$

(2) $$F\left(\beta \right)=\frac{X_{\beta }}{X_{\beta }+X_{\alpha }}=\frac{A_{\beta }}{\left[\left(\frac{K_{\beta }}{K_{\alpha }}\right)A_{\alpha }+A_{\beta }\right]}$$

where *I*_1250_ and *I*_1231_ represent the peak areas at 1250 and 1231 cm^−1^, respectively. The *A*_α_ and *A*_β_ represent the absorbance at 765 cm^−1^ and 840 cm^−1^, the *K*_β_ and *K*_α_ are the absorption coefficient at the respective wavenumbers, which are 7.7 × 10^4^ and 6.1 × 10^4^ cm^2^ mol^−1^ in value [36]. Figure 2j(I) shows the FTIR magnification of PAN and PAN/PVDF in the band 1200–1300 cm^−1^, and Figure 2j(II) shows PVDF and PAN/PVDF in the band 740–860 cm^−1^. According to the calculation, the C value and F(β) value of PAN/PVDF are 1.0541 and 2.078, respectively, which are higher than those of pure PAN (C = 0.9852) and pure PVDF (F(β) = 0.498). The result indicates that the piezoelectric properties of PAN/PVDF composite films are better than those of single content films with PAN or PVDF alone.

Figure 2k shows the XRD patterns of PAN, PVDF and PAN/PVDF, where two typical diffraction peaks at 17° and 20° are notable. The former corresponds to the (100) crystal plane diffraction peak of PAN, and the latter corresponds to the PVDF β phase diffraction at (110/200) plane [38,39]. To sum up, the doping of PAN and PVDF can increase the content of their planar zigzag conformation and β phase, thus improving their piezoelectric properties. This further verified the enhanced effect of the composite with PAN and PVDF on the piezoelectric properties.

### 3.3. Piezoelectric Output of FIPS

**Figure 3:**
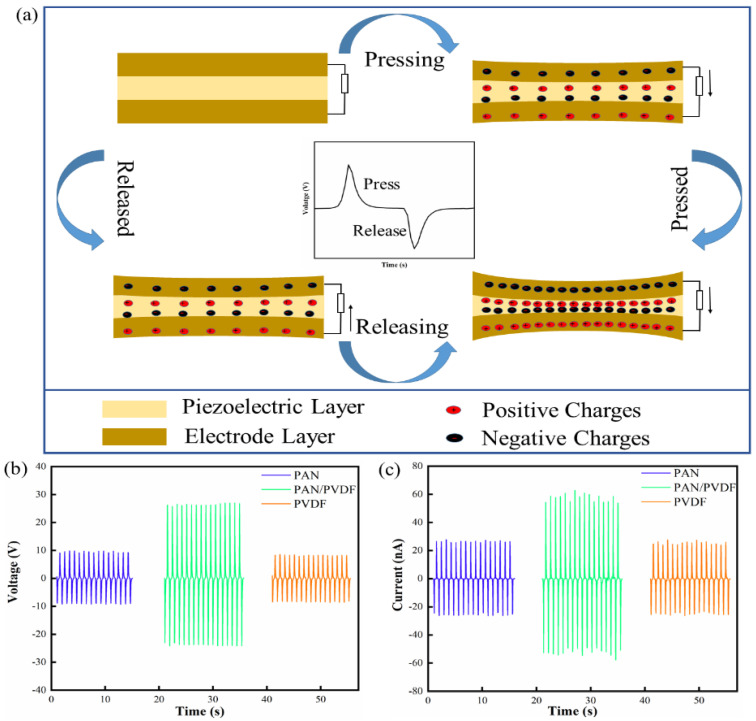
(**a**) Schematic illustration of the FIPS working process. (**b**,**c**) Piezoelectric output performance of the PAN composite film, PAN/PVDF composite film and PVDF composite film.

The piezoelectric output mechanism of the FIPSs is shown in Figure 3a, and each cycle generation is divided into four processes. The FIPS is deformed under the action of external stimulation, resulting in a potential difference between the upper and lower electrodes. With the disappearance of stimulation, the potential difference between the two ends gradually decreases to zero, and the FIPS returns to the initial uncharged state. At the same time, the polarity of the charge changes with the change of the direction of external stimulation. In order to understand the piezoelectric effect more intuitively, the illustration in Figure 3a shows the formation of a piezoelectric voltage output waveform.

Here, a self-made test platform is used to provide a reciprocating pressure that can be accurately controlled by the linear motor. It is worthy of note that triboelectricity generated by the surface contact between two different materials can interfere with the electrical signal of FIPS [40,41]. To avoid this effect, the top and bottom sides of the FIPS are encapsulated, and the measuring instrument with polyimide film is designed with the same surface material. Moreover, an effort is made to reduce the distance between the force supply device connected to the linear motor and the FIPS.

**Figure 4:**
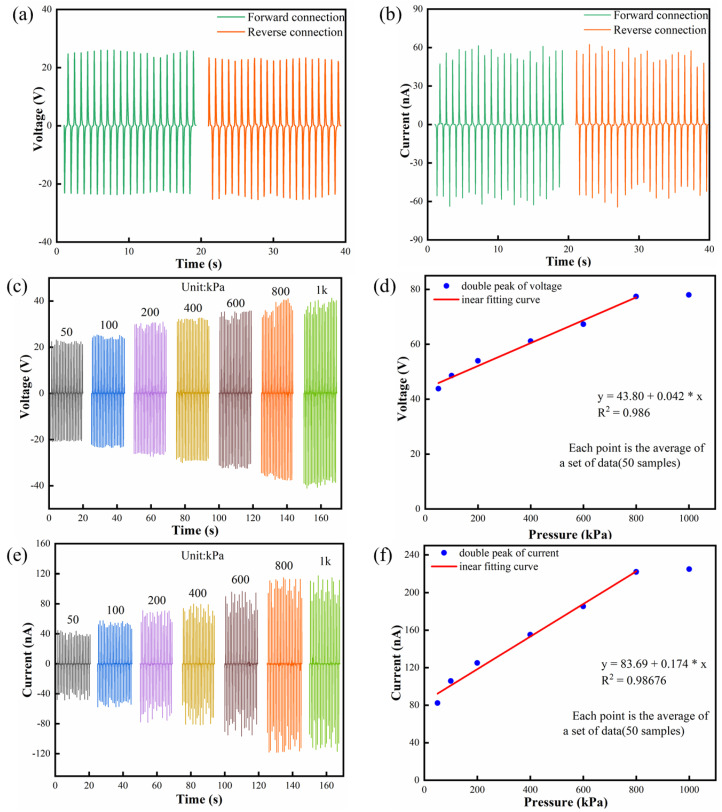
(**a**,**b**) The output voltage/current of the FIPS with forward and reverse connection modes. (**c**,**e**) The output voltage/current under different applied pressures. (**d**,**f**) The linear fitting analysis calculated from (**c**,**e**).

The output voltages of different FIPS devices using PAN, PVDF and PAN/PVDF, shown in Figure 3b, are measured by continuous compression shock (10 N) onto an effective area of 1 cm × 1 cm at a frequency of 1 Hz and a load resistance of 600 MΩ. (Note: all the following tests are carried out under this condition). The corresponding voltage values are 19 V, 16 V and 49 V for the above three types of devices, respectively. Figure 3c shows that the output currents of the FIPS with PAN/PVDF is about 2 times and 2.2 times that of the FIPSs with PAN and PVDF, respectively. Obviously, the electrical properties of the FIPS are significantly enhanced by the way of material compositing of PAN/PVDF. This is mainly induced by the content increase of the planar zigzag conformation and β phase in mutual doped PAN and PVDF powders and results in a great improvement in their piezoelectric properties. This can be taken as a typical synergistic piezoelectric effect. The conclusion is consistent with the results of previous FTIR and XRD analysis. In addition, the FIPS has a completely symmetrical structure, which produces almost the same piezoelectrical signal output when connected in the forward or reverse direction. Figure 4a,b show the classic switch polarity test results that demonstrate that the output voltage and current are basically reversible in both forward and reverse connection modes, indicating a pure piezoelectric signal [42].

The sensitivity and linearity are important static parameters in judging the performance of the sensor. When the FIPS is used to identify human posture and motion, stable piezoelectric output signals are needed under variable tension and pressure. In order to verify the feasibility of the FIPSs as a pressure sensor, a linear motor is used to simulate periodic pressure, thus the mathematical model of the relationship between output performance and pressure of the FIPSs is established to test the sensing characteristics. Figure 4c,e presents the change relationship of peak-to-peak voltage and current of the FIPS with increasing pressure from 50 kPa to 1000 kPa. It can be clearly observed that there is an obvious linear growth relationship between the output performance of the FIPS and the pressure changing from 50 kPa to 800 kPa, and then it gradually reaches a relatively saturated state from 800 to 1000 kPa. As shown in Figure 4d, the linear regression equation of the peak-to-peak voltage of the FIPS is: y = 43.8 + 0.042x, where y is the output voltage of the FIPS and x is the pressure on the FIPS. Therefore, the fitting slope of the voltage–pressure curve of the FIPS in the range of 50 kPa to 800 kPa is 42 mV/kPa (voltage sensitivity) and the voltage linearity is 0.986. In Figure 4f, the linear regression equation of the peak-to-peak current of the FIPS is: y = 83.69 + 0.174x, and the current sensitivity and linearity of the FIPS are 0.174 nA/kPa and 0.98676, respectively. Yang et al. reported a piezoelectric sensor based on ZnO/PVDF, whose pressure sensitivity was 3.12 mV/kPa [21]. Similarly, Liu et al. fabricated a piezoelectric tactile sensor, and the slope of the voltage−pressure curve indicated a sensitivity of 7.1 mV/kPa [13]. Zhu et al. developed a hybrid pressure sensor with a sensitivity of 0.06 V/N (6 mV/kPa) [43]. Compared with them, the fabricated FIPSs have an obvious advantage. In general, the pressure range of normal people is 0 kPa–370 kPa, and the foot pressure produced by human body weight and posture movement is more than 100 kPa [44]. The output signal of the FIPS has excellent linearity and sensitivity in the pressure range of 50 kPa to 800 kPa, which covers the pressure caused by various posture movements of the human body. This indicates that the designed FIPSs can reliably identify various action information of the human body and convert it into electrical signals to realize the functions of human posture monitoring and gait recognition.

**Figure 5:**
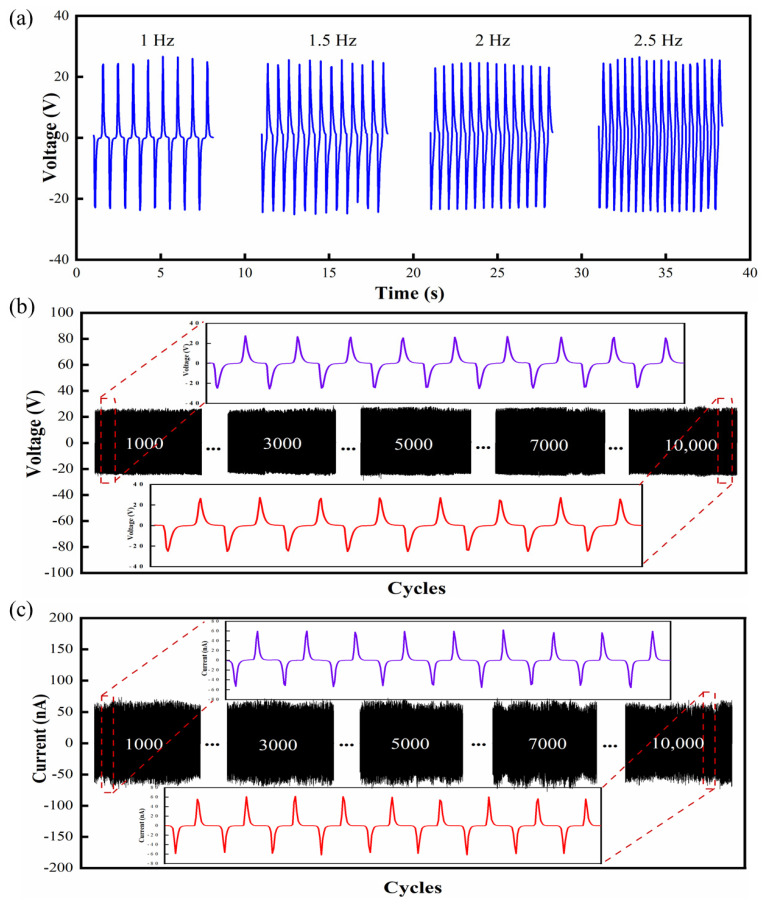
(**a**) The frequency response of the FIPS (1 Hz-2.5 Hz). (**b**,**c**) Voltage/current stability test of the FIPS (inset shows the enlarged view at 10^3^ cycles and 10^4^ cycles).

Stability and response time are two other key factors influencing sensor reliability. Figure 5a shows the output voltage of the FIPS at different frequencies. When the working frequency is raised from 1 Hz to 2.5 Hz of typical human posture motion, the change of frequency has almost no obvious effect on the output voltage. In terms of theoretical analysis, the output voltage of the FIPS can be calculated by this equation.

(3) $$U=\frac{Q}{C}=\frac{d_{33}Fh}{\varepsilon_{0}\varepsilon_{r}A},$$

where *d_33_* represents the piezoelectric strain coefficient, *F* represents the mechanical stress, *h* and *A* represent the thickness and area of the piezoelectric device, and *ε_0_* and *ε_r_* represent the vacuum dielectric constant and the relative dielectric constant of the piezoelectric material, respectively. Therefore, the output voltage of the FIPS is not related to the frequency, which is consistent with the experimental results. Generally, the frequencies of a person walking normally, jogging, jumping and running are 1 Hz, 1.6 Hz, 1.8 Hz and 2.3 Hz [45], respectively, which is within the frequency range of the output voltage at the different frequencies measured earlier. This implies that the device can maintain good stability under the regular human posture and movement environment. Furthermore, the voltage and current stability of the FIPS was tested under cycle pressure at 1 Hz working frequency. As can be seen from Figure 5b,c there was no obvious decrease after 10,000 cycles and it still had good stability. In detail, the enlarged view extracted from the data of 1000 cycles and 10,000 cycles, respectively, showed that these wave forms keep the same. In addition, the response time of the FIPS decreases with the increase of frequency (Figure S3), the minimum response time is 43 ms at the frequency range of 1–2.5 Hz. This indicates that the FIPSs can satisfy the requirement of fast response in human-motion detection. Furthermore, the output performance of the pre-prepared FIPS was re-measured a month later, as shown in Figure S4. Compared with the output performance of the FIPS measured for the first time, the output voltage and current of the FIPS remained almost unchanged after one month. This information about reproducibility proves that the FIPS has good stability and can be used for a long time. The experimental results proved that the FIPS have obvious advantages of electrical performance and mechanical reliability, while its adaption will be further verified in practical measurement.

### 3.4. Recognition of Human Posture and Movement Signals

**Figure 6:**
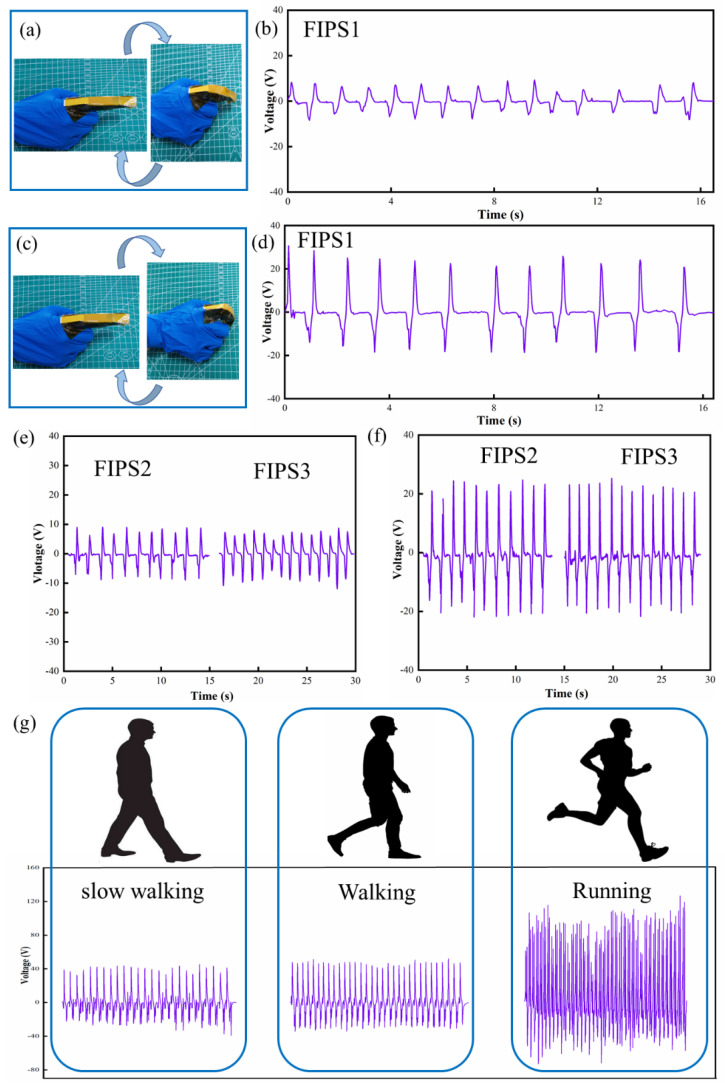
Application of the FIPS for human body pressure recognition. (**a**,**c**) The FIPS was placed on the finger. The output voltage of three FIPSs when the fingers are bent at (**b**,**e**) 20 degrees and (**d**,**f**) 45 degrees. (**g**) The FIPS output signal in three kinds of exercise states (slow walking, walking and running).

More advantages of the fabricated FIPS are its light weight, self-powered supply, flexibility and wide measuring range (0–800 kPa), which make more applications, including human action recognition and posture detection, possible. When the FIPS is tied on various joints of the human body, the joint motion causes the FIPS deformation including bending, stretching and extrusion, resulting in piezoelectrical signal output reflecting motion state at the same time. As shown in Figure 6a,c, the FIPS is encapsulated in polyimide films and then attached to the finger joint. Figure 6b shows the voltage output is about 8.66 V and the response time is 68 ms when the finger is bent 20°. Similarly, Figure 6d presents the voltage/response time change of about 22.5 V/86 ms at 45°. By comparison, when the fingers are bent at different angles, the piezoelectric output signal of the FIPS will be obviously different (Video S1). Moreover, the other two FIPSs are attached to the same finger joint in the same way, and the output voltage of the FIPSs is measured when the finger is bent 20° and 45° (Figure 6e,f). By comparing the Figure 6e,f with the Figure 6b,d, it can be seen that the output voltage of the three FIPSs are basically the same when the fingers are bent 20°/45°. This is because when the same finger bends at the same angle, the deformation caused by the FIPS is basically the same, so the output voltage of the three FIPSs remains the same. This proves that the prepared FIPSs have good consistency and stability. For human posture detection, the FIPS is fixed on the soles of the feet, and are selected to identify different gaits such as slow walking, walking and running. The practical measurement is then carried out. As shown in Figure 6g (Videos S2 and S3), three gaits can be clearly distinguished due to their significant difference in voltage and frequency. The extracted voltage corresponding to slow walking, walking and running is 38.9 V, 40.1 V and 87.4 V, respectively, and the corresponding frequencies is 1 Hz, 1.6 Hz and 2.3 Hz, respectively.

From the actual research results, it can be seen that the well-designed FIPSs have a wide measurement range (0–800 kPa), which covers the pressure caused by various posture movements of the human body, so the FIPSs can be used for human posture recognition. At the same time, the FIPSs have good external perception and high output performance, which can convert multiple joint motions and different human motions into easy to obtain electrical signals, and realizes posture detection and gait recognition by distinguishing different electrical signals. Furthermore, the FIPSs have good stability and durability, and have not been damaged after bring used many times. The prepared FIPS still has excellent output performance after being used for one month. Hence, the FIPS has broad application prospects for wearable electronic products, and are of great significance for human–machine interaction, medical testing and personalized recognition.

## 4. Conclusions

In summary, we fabricated a unique FIPS based on a PAN/PVDF composite film with excellent flexibility, stretchability and electrical performance, by combining piezoelectric and conductive particles into solid silicone rubber. Due to the rationality of the preparation process and the superiority of the materials, the FIPSs achieved integration and exhibited good electrical output and mechanical durability without being damaged in more than 10,000 compression and bending cycle tests. Meanwhile, the output performance of the FIPS does not decline significantly after long-term placement. Moreover, by reason of the synergistic piezoelectric effect of PAN and PVDF, its piezoelectric properties are greatly improved, which sconvert different posture movements of the human body into electrical signals that that can be easily obtained and observed. Under the action of external pressure, the developed FIPS has good sensitivity (voltage = 42 mV/kPa, current = 0.174 nA/kPa), high linearity (voltage = 0.986, current = 0.98676) and a wide measuring range (0–800 kPa). These excellent sensing properties enable the FIPSs to respond to different external stimuli in terms of degrees of freedom, strain rate and strain frequency. Moreover, the FIPSs showed good stability and consistency, and the output performance of multiple FIPSs is basically the same under the same pressure. The experimental and practical measurements showed that the proposed pressure sensor based on PAN, PVDF, silicone rubber and silver-coated glass microspheres is very suitable for human posture detection and motion recognition. This study aims to explore a new and feasible method to improve the performance of flexible pressure sensors for application in wearable electronics, and providing a superior flexible device for human–machine interaction, medical testing and personalized recognition.

## Data Availability

Data is contained within the article and the Supplementary Materials.

## Supplementary Materials

The following supporting information can be downloaded at: https://www.mdpi.com/article/10.3390/nano12071155/s1, Figure S1: Data collection and analysis interface diagram; Figure S2: Energy dispersive spectroscopy (EDS) spectrum of the PAN/PVDF composite film; Figure S3: The response time of the SFPS under an applied pressure of 10N at different frequencies. Figure S4: The output performance of the FIPS after one month. (a) Voltage. (b) Current. Table S1: All the abbreviations used in the manuscript. Video S1: Real-time monitoring of finger movement by the FIPS. Video S2: Real-time monitoring of human walking movement by the FIPS. Video S3: Real-time monitoring of human running movement by the FIPS.
