# Supplementary material for: Synergistic Enhancement Properties of a Flexible Integrated PAN/PVDF Piezoelectric Sensor for Human Posture Recognition
Source: Nanomaterials (Basel). 2022 Mar 31;12(7):1155. doi: 10.3390/nano12071155 (PMC9000213; doi:10.3390/nano12071155)
Supplement: Supplementary file 1 [file nanomaterials-12-01155-s001.zip › nanomaterials-1650557-supplementary.pdf]

Supporting Information

Synergistic enhancement properties of flexible integrated PAN/PVDF piezoelectric sensor for human posture recognition

Jiliang Mu *, Shuai Xian, Junbin Yu, Juanhong Zhao, Jinsha Song, Zhengyang Li, Xiaojuan Hou, Xiujian Chou and Jian He *

Science and Technology on Electronic Test and Measurement Laboratory, North University of China, Taiyuan 030051, China

Email: mujiliang@nuc.edu.cn (Jiliang Mu),drhejian@nuc.edu.cn (Jian He)

Keywords: PAN/PVDF; integrated structure; synergistic piezoelectricity; flexible pressure sensor; human posture recognition

Table S1. All the abbreviations used in the manuscript

full title	abbreviation
polyacrylonitrile	PAN
polyvinylidene fluoride	PVDF
flexible integrated pressure sensor	FIPS
piezoelectric nanogenerator	PENG

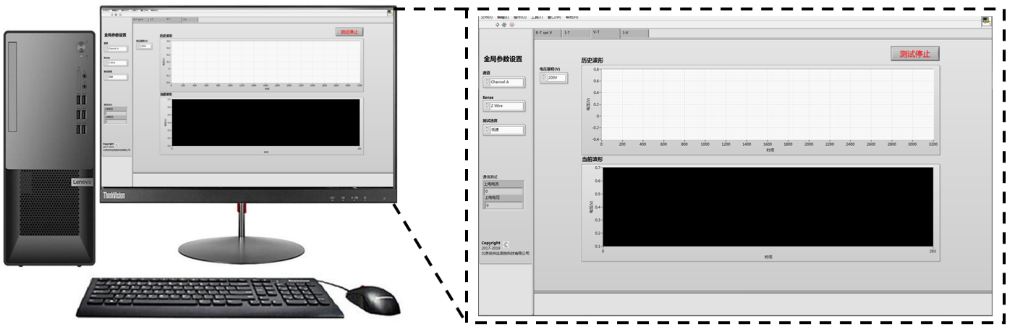

Figure S1. Data collection and analysis interface diagram.

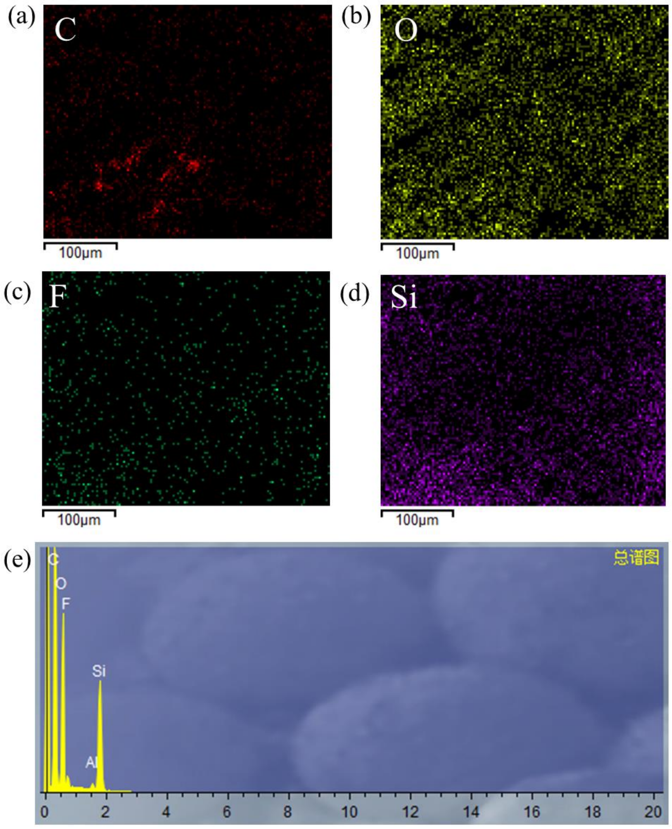

Figure S2. Energy dispersive spectroscopy (EDS) spectrum of the PAN/PVDF composite film.

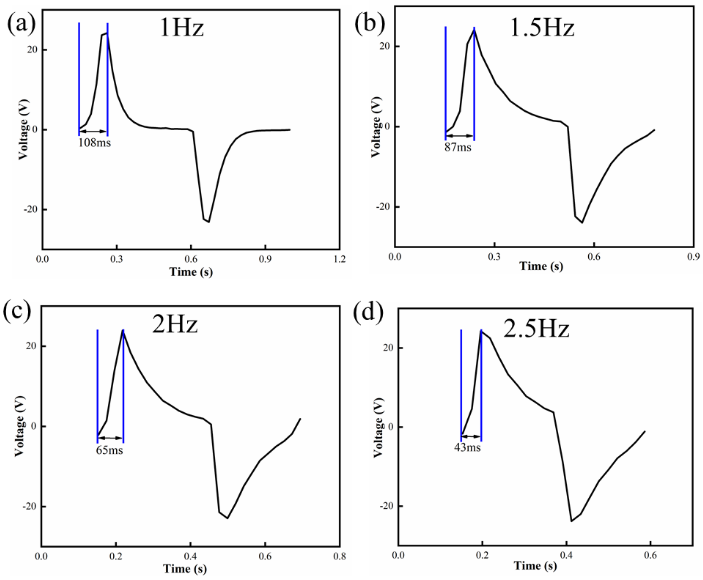

Figure S3. The response time of the SFPS under an applied pressure of 10N at different frequencies.

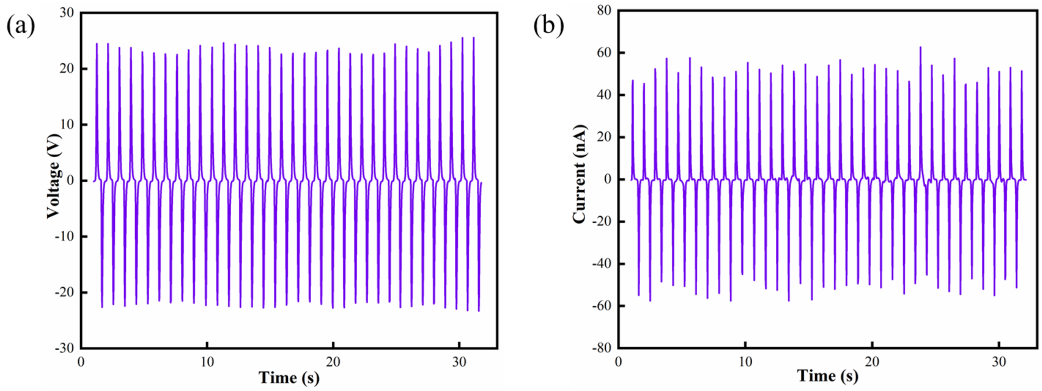

Figure S4. The output performance of the FIPS after one month. (a) Voltage. (b) Current.
